# Supplementary material for: Fast and robust metagenomic sequence comparison through sparse chaining with skani
Source: Nat Methods. 2023 Sep 21;20(11):1661–5. doi: 10.1038/s41592-023-02018-3 (PMC10630134; doi:10.1038/s41592-023-02018-3)
Supplement: Supplementary file 1 — Supplementary Note, Figs. 1–12 and Tables 1–3. [file 41592_2023_2018_MOESM1_ESM.pdf]

nature methods

Brief Communication

https://doi.org/10.1038/s41592-023-02018-3

Fast and robust metagenomic sequence comparison through sparse chaining with skani

In the format provided by the authors and unedited

Supplementary Note

Cophenetic correlation information

To quantify the goodness of cluster heatmaps in Fig. 1 and Extended Data Fig. 4, we use cophenetic correlation [1] for the associated dendrograms. Given any hierarchical clustering dendrogram and any distance matrix, cophenetic correlation gives a value from -1 to 1, with higher values indicating stronger concordance between the dendrogram and matrix. Importantly, cophenetic correlation takes into account branch lengths in the dendrogram, unlike the commonly used unweighted Robinson-Foulds distance [2] between trees. We evaluate each method's dendrogram, obtained by average-linkage clustering from its *own distance matrix*, against *ANIm's distance matrix*. As a baseline, we take the correlation between ANIm's dendrogram against its own distance matrix. Note that even ANIm's cophenetic correlation against its own distance matrix may not be equal to 1.

Method (version)	Commands used (arguments in parenthesis)
skani (v0.1.0)	`skani sketch (dataset) -o (sketches) -t 50; skani search -d (sketches) (query genome) -t 50; skani dist -q (query genome) -r (sketches)/* -t 50; skani triangle (genomes)`
Mash (v2.3)	`mash sketch (dataset) -o (sketches) -p 50; mash dist (query genome) (sketches) -p 50`
FastANI (v1.33)	`fastANI --rl (dataset) -q (query genome) -t 50`
sourmash (v4.5)	`sourmash sketch dna --output-dir (sketches) (dataset); sourmash compare (sketches)/*.sig -k 31 --max-containment --ani`
ANIu (v1.2)	`java -jar OAU.jar -n 20 -f1 (genome 1) -f2 (genome 2) -u (usearch binary)`
ANIm (pyani v0.2.12)	`average_nucleotide_identity.py -m ANIm -i (genomes) -o (output folder) --workers 50`

Supplementary Table 1: Method commands and parameters used for benchmarking.

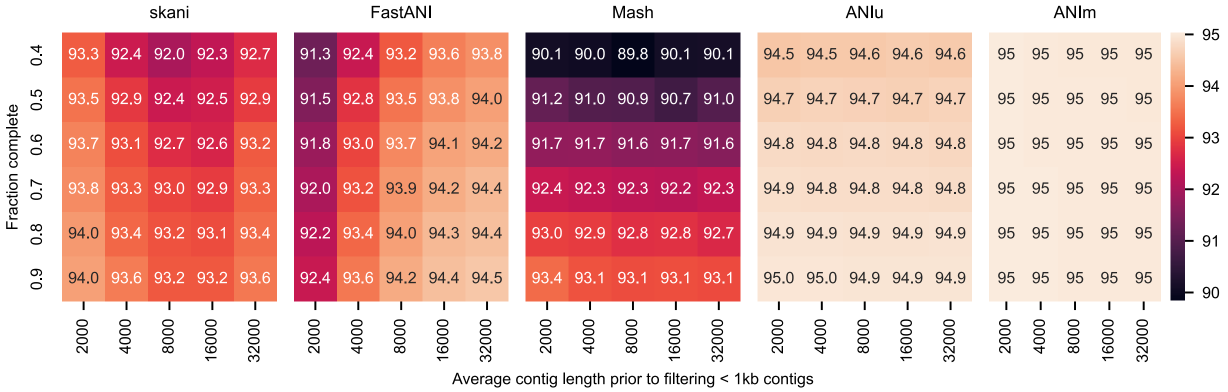

Supplementary Fig. 1: We performed the same simulation procedure as in Extended Data Fig. 1 and additionally induced random point substitutions so that the true pairwise ANI is 95%. Notably, skani underestimates ANI on the simulated dataset whereas on real datasets slight overestimation of ANI is more common (Supplementary Fig. 6). This likely stems from skani's heuristic for dealing with fragmented assemblies, which removes non-homologous k-mers only on the ends of the chunks (see **Estimating ANI from chains** in Methods), not being as effective on i.i.d random fragments between two genomes. Importantly, fragmentation in real assemblies depends on the genomic loci characteristics (e.g. sequence repetitiveness), so is not i.i.d between two genomes.

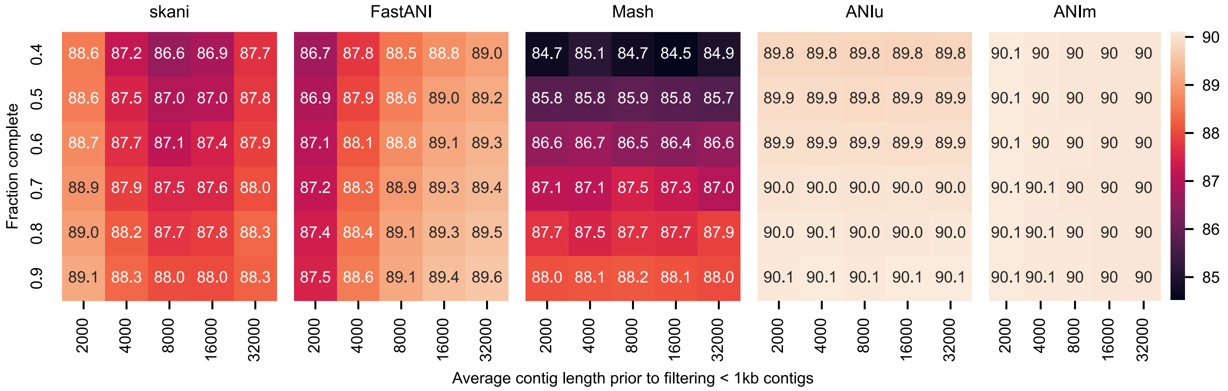

Supplementary Fig. 2: We performed the same simulation procedure as in Extended Data Fig. 1 and additionally induced random point substitutions so that the true pairwise ANI is 90%.

dataset name	Description	Query genome(s)	Availability
Pasolli et al 25-50	All MAGs in Pasolli et al [3] in species-level bins between 25-50 genomes (8611 genomes)	all-to-all comparisons within each bin	`http://segatalab.cibio.unitn.it/data/Pasolli_et_al.html`
Ocean archaea MAGs	4435 archaea MAGs in non-representative OceanDNA MAGs from Nishimura and Yoshizawa [4] with $>$ 90% ANI (according to skani)	all-to-all	`https://doi.org/10.6084/m9.figshare.c.5564844.v1`
Soil MAGs	1859 soil prokaryotic MAGs from soil genomes collected in Olm et al [5] with $>$ 90% ANI (according to skani)	all-to-all	`https://figshare.com/collections/Genomes_for_consistent_metagenome-derived_metrics_verify_and_define_bacterial_species_boundaries/4508162/1`
Ocean eukaryotic MAGs	982 eukaryotic MAGs from Alexander et al [6] and 713 eukaryotic MAGs from Delmont et al [7] with $>$ 90% ANI (according to skani)	all-to-all within each dataset	`https://osf.io/gm564/` and `https://www.genoscope.cns.fr/tara/localdata/data/SMAGs-v1/SMAGs_contigs_individual.fna.tar.gz`
Nayfach MAGs	52,515 MAGs from Nayfach et al [8]	all-to-all	`https://genome.jgi.doe.gov/GEMs`
E. coli genomes	4350 *E. coli genomes.* dataset D3 from Jain et al. [9]).	E. coli K12 (NC_007779)	dataset D3 from `http://enve-omics.ce.gatech.edu/data/fastani`.
B. anthracis genomes	571 *B. anthracis genomes.* dataset D2 from Jain et al. [9]).	Bacillus anthracis 52-G (NZ_CM002395.1)	dataset D2 from `http://enve-omics.ce.gatech.edu/data/fastani`.
refseq-rc (representative and complete)	4233 complete/chromosome level representative assemblies from refseq. Downloaded Sept. 16, 2022	all-to-all	`ncbi-genome-download --assembly-levels complete,chromosome --refseq-categories representative --formats fasta bacteria,viral,archaea,fungi` , `https://zenodo.org/record/8058221`
GTDB	65703 genomes from the GTDB database [10], release 207.	E. coli K12 (NC_007779)	Download at `https://gtdb.ecogenomic.org/`
Parks MAGs	7901 MAGs from Parks et al. [11].	Pseudomonas stutzeri (GCA_002292085_1) MAG (also from Parks et al. [11])	dataset D5 from `http://enve-omics.ce.gatech.edu/data/fastani`.
B. fragilis genomes	318 *B. fragilis* genomes from refseq.	Bacteroides fragilis YCH46 (NC_006347.1)	`ncbi-genome-download --genera "Bacteroides fragilis" bacteria --formats fasta`, `https://zenodo.org/record/8058221`

Supplementary Table 2: Extended description of datasets used in Fig.1 and three additional datasets (Parks MAGs, *B. anthracis* genomes, and *B. fragilis* genomes) with additional plots shown in Supplementary Fig. 7. The refseq-rc and *B. fragilis* datasets were generated using ncbi-genome-download from `https://github.com/kblin/ncbi-genome-download`. and available from `https://zenodo.org/record/8058221`.

dataset	Size of index on disk
E. coli genomes (4350 genomes)	4.1 GB
GTDB (65,703 genomes)	40 GB
refseq-rc (4233 genomes)	3.0 GB

Supplementary Table 3: Size of stored index (i.e. sketches) on disk for three datasets.

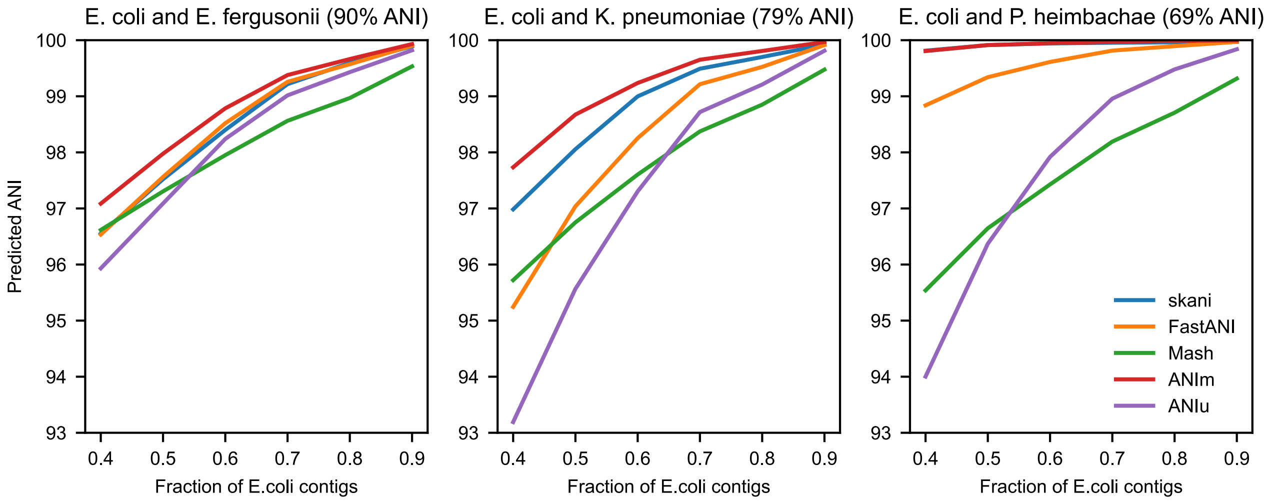

Supplementary Fig. 3: We simulated a chimeric MAG by sampling 15kb chunks of an *E. coli* genome and a *E. fergusonii, K. pneumoniae*, or *P. heimbachae* genome (with pairwise ANIu ANI in title) where the x-axis indicates the probability of sampling from the *E. coli* genome, i.e. the fraction of *E. coli* present in the MAG. The predicted ANI for each method between the original *E. coli* genome and the generated chimeric MAG is plotted. The lower ANI for ANIu is due to its sensitivity (i.e. ability to compare low ANI genomes), causing the contamination due to chimericism to lower the predicted ANI.

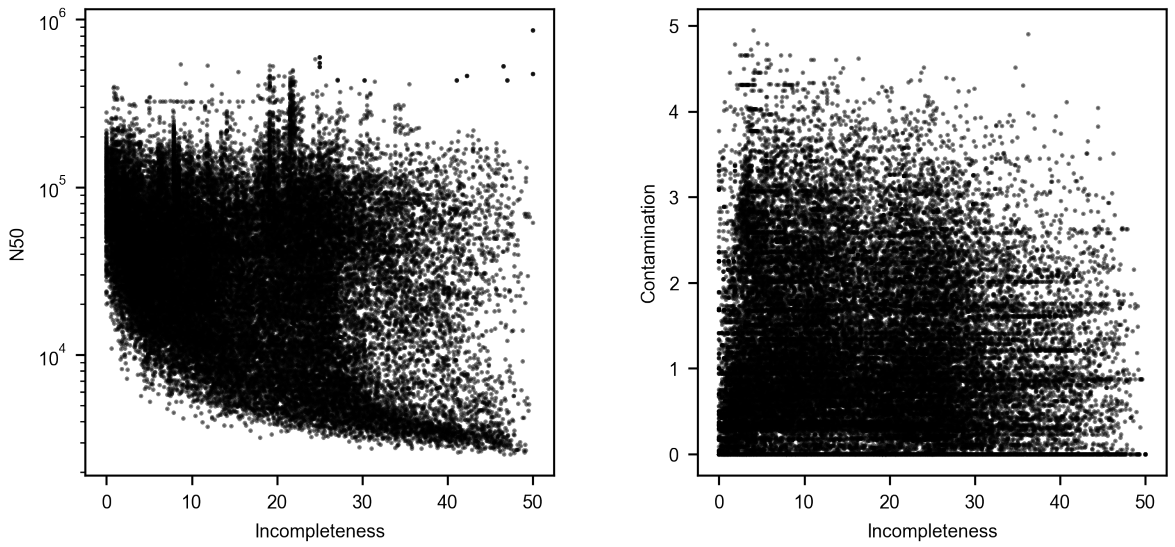

Supplementary Fig. 4: Scatter plot of the data points, representing pairs of genomes, in Fig. 1b and Extended Data Fig. 3.

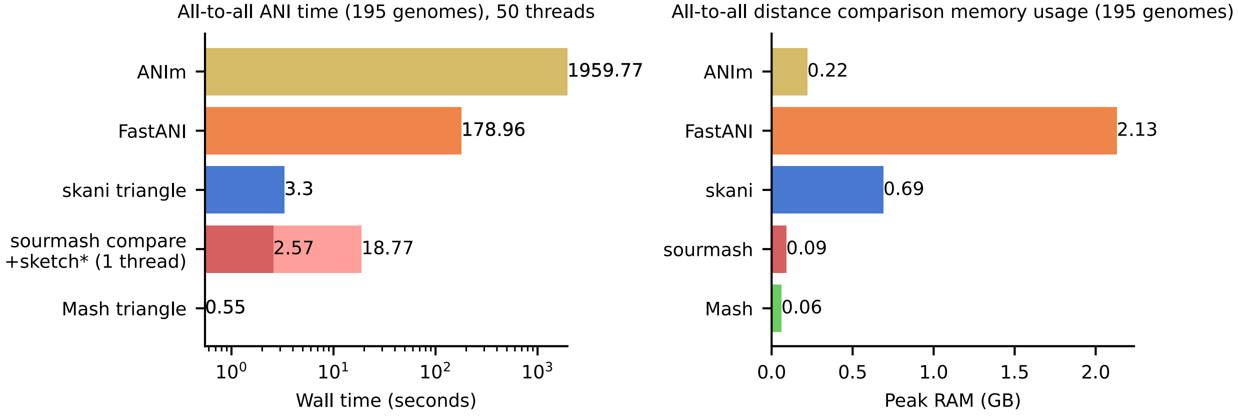

Supplementary Fig. 5: Running times and memory usage for all-to-all comparisons on the 195 genomes in Fig.1c with 50 threads. sourmash's latest release did not have multi-threading support so sourmash was run with one thread. Out of sourmash's 18.77 seconds runtime, 2.57 was used for ANI computation and the rest was for sketching.

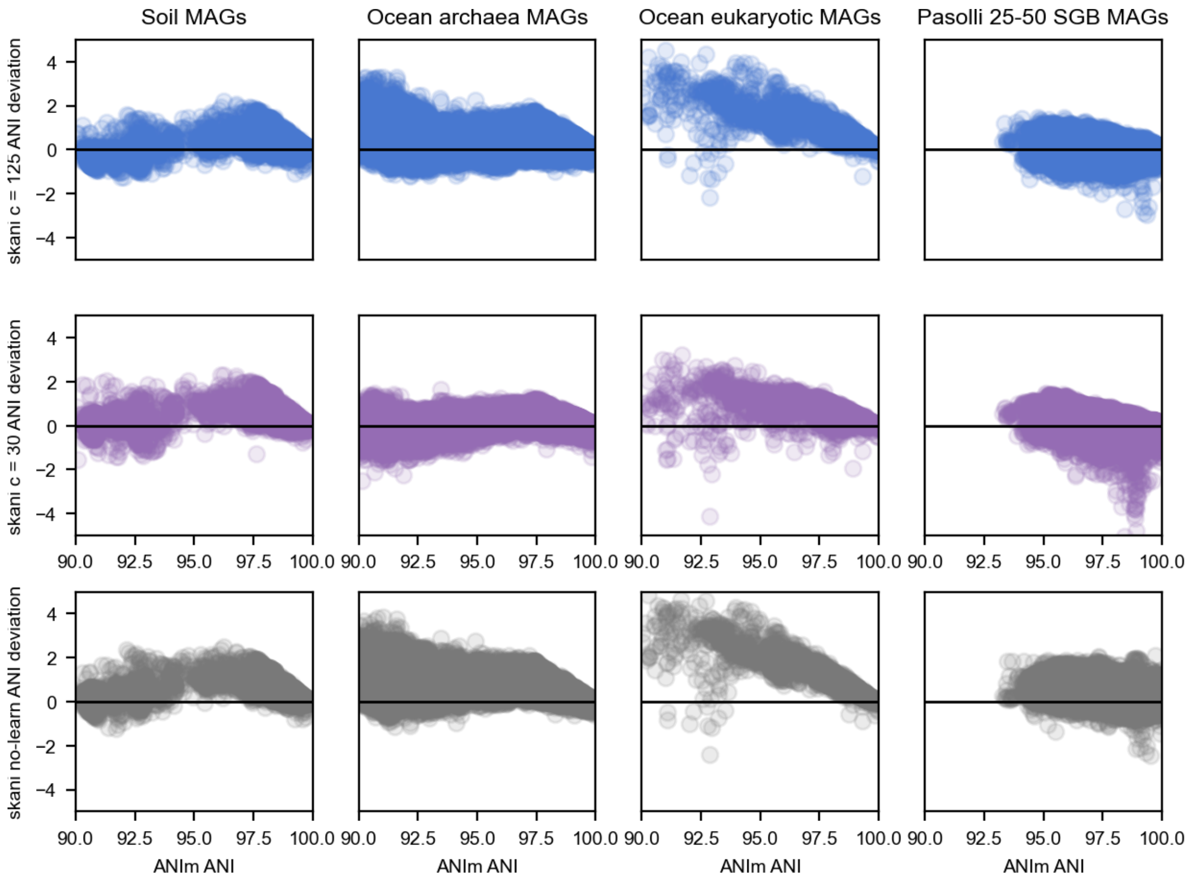

Supplementary Fig. 6: skani's ANI deviation from ANIm as a function of ANI. On the eukaryotic and archaea datasets, lowering c visibly decreases the bias, especially for smaller ANI values close to 90%. However, it has a smaller effect on the soil and Pasolli datasets. skani no-learn corresponds to default parameters ($c = 125$) without the learned ANI regression.

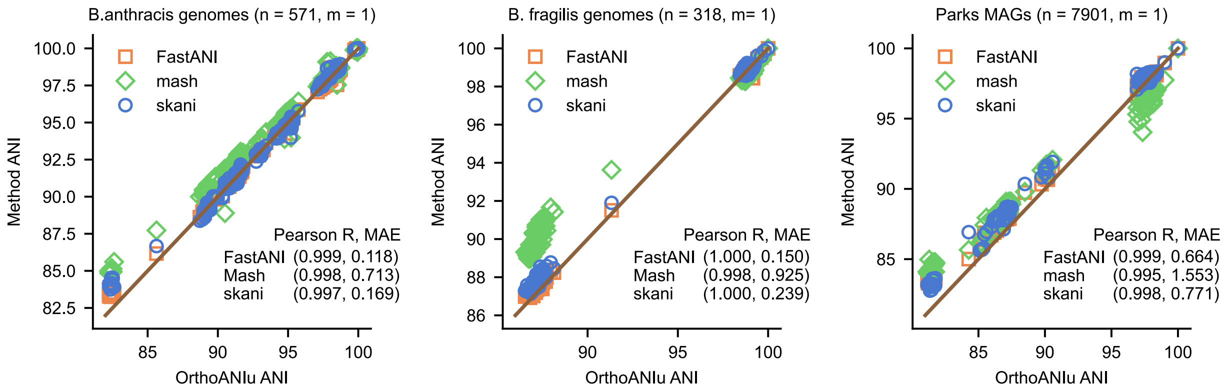

Supplementary Fig. 7: Additional ANI experiments on three different datasets specified in Supplementary Table 2. A single query genome (m = 1) was queried against n reference genomes. The same metrics and methodology was used as in Fig. 2a.

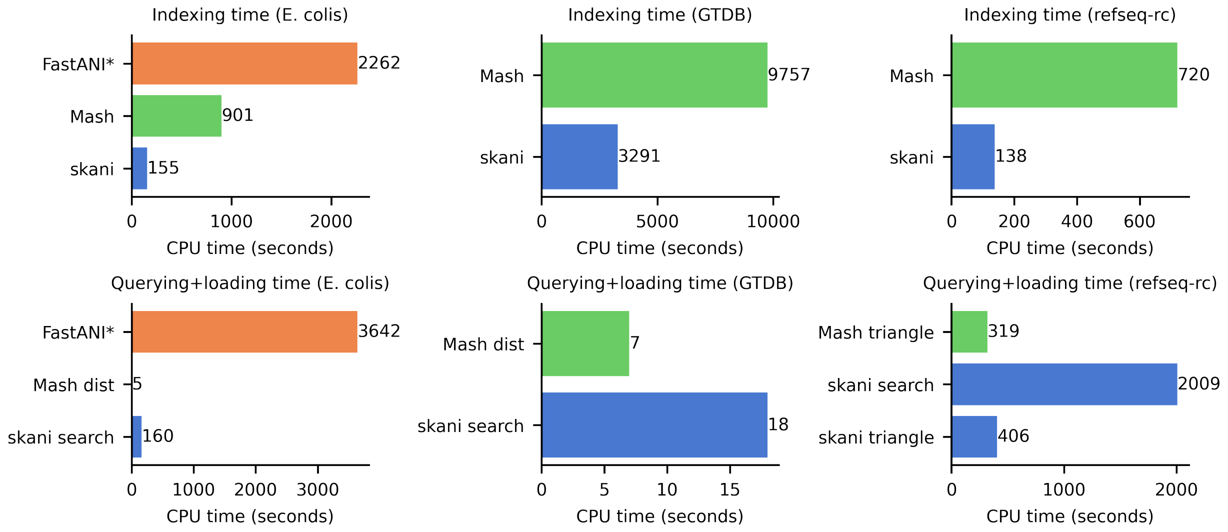

Supplementary Fig. 8: Benchmarking times for the three experiments run in Fig. 2 timed in CPU time. The exact same experiments were run, only with CPU times shown instead of wall times.

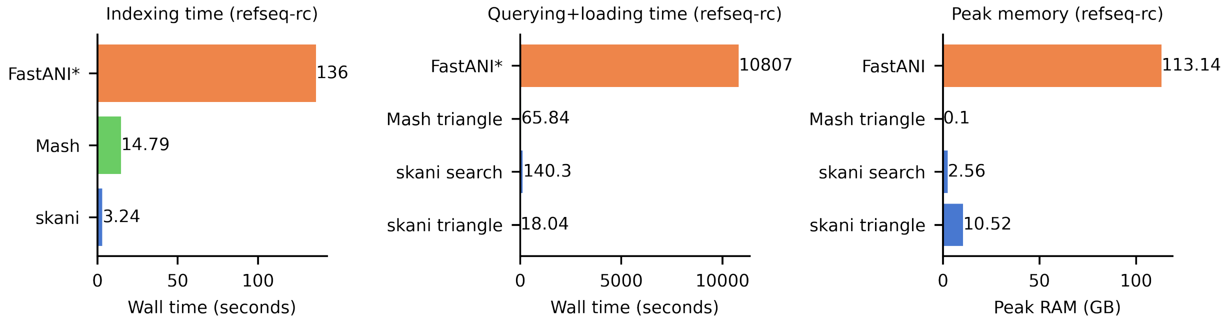

Supplementary Fig. 9: The same runtime as in Fig. 2 for the refseq-rc dataset but with FastANI's runtimes included. The timing comparison is unfair to FastANI because FastANI computes ANI for genomes as low as 75% ANI in practice and is more sensitive than skani, which uses an 80% putative ANI filter by default.

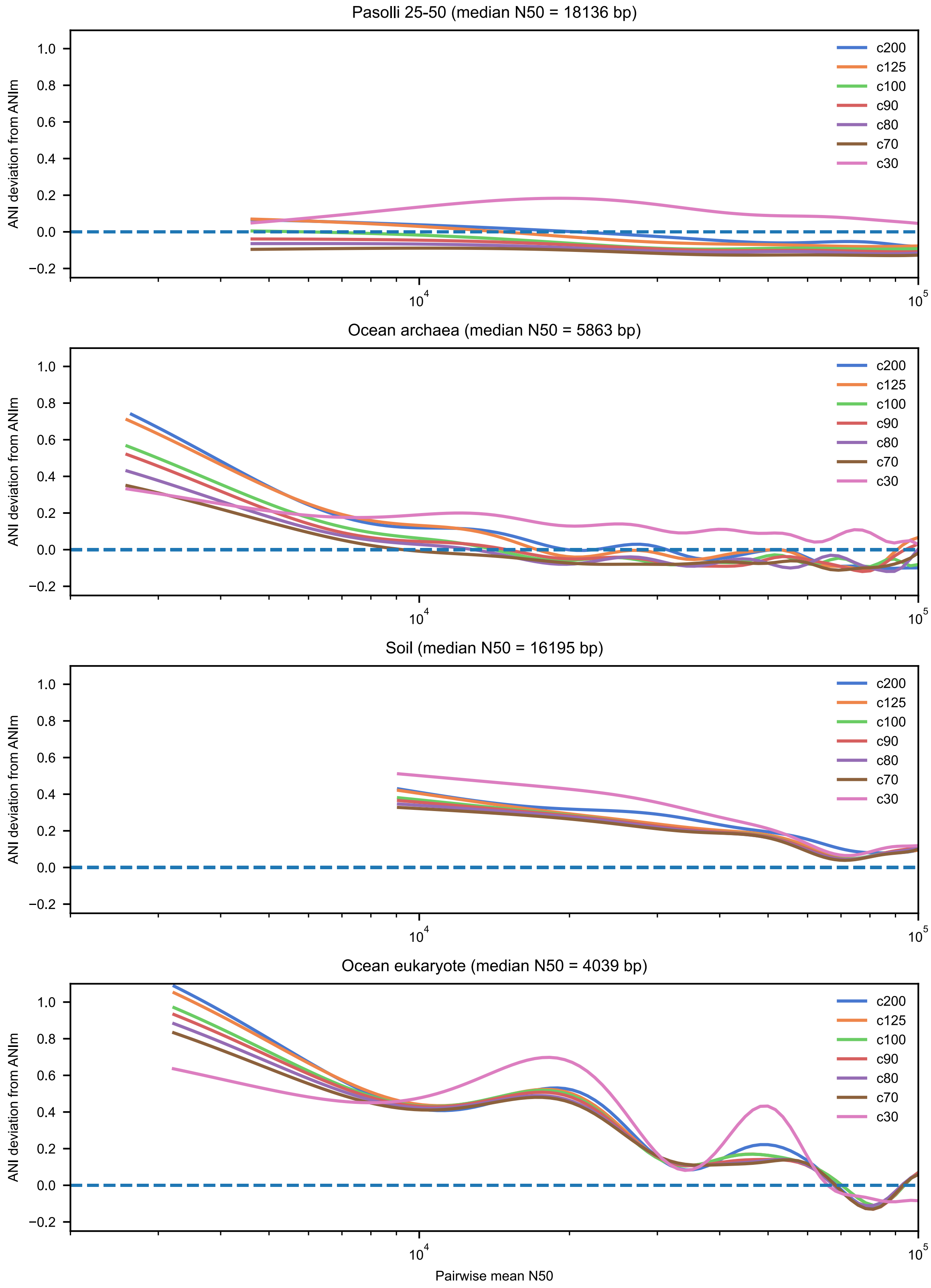

Supplementary Fig. 10: skani's deviation from ANIm as a function of N50 with varying values of c. Regression lines were obtained by subsampling 10000 calculations and using a SVM regression with a RBF kernel as implemented by sklearn [12] v0.22.1. Note that by default skani's learned ANI regression is turned off for $c = 30$, but turned on otherwise.

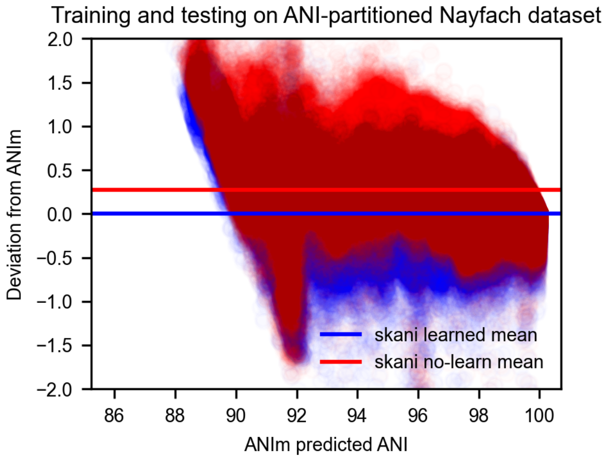

Supplementary Fig. 11: We partitioned the Nayfach et al dataset into two equal parts such that skani has no predicted ANI (i.e. AF $< 15\%$) for any two MAGs across the partition. We trained on one of the parts when the ANIm ANI is $> 90\%$, and tested on the other. Each dot represents skani's deivation from ANIm's ANI for one pair of genomes. Horizontal lines indicate the mean deviation over all comparisons.

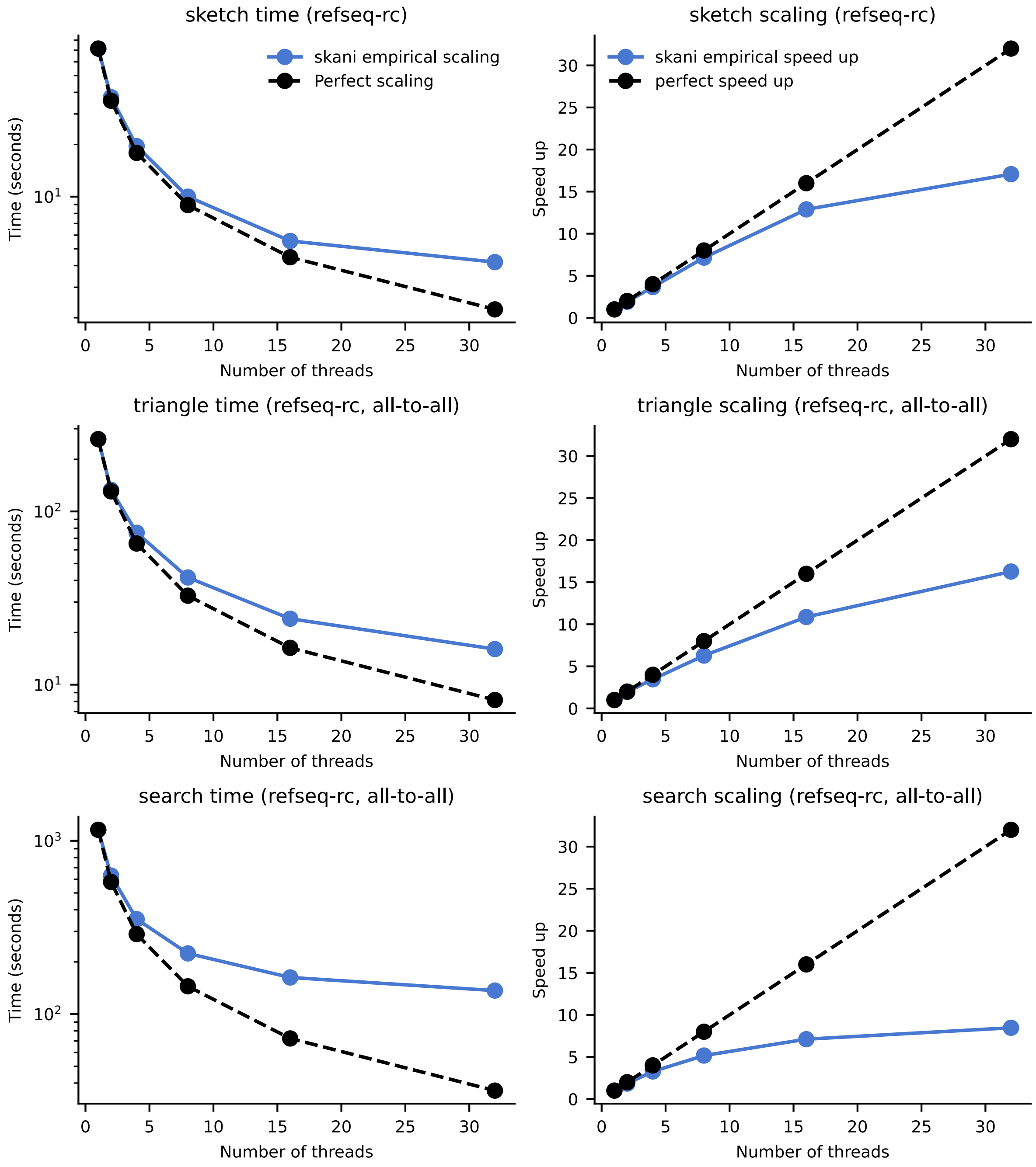

Supplementary Fig. 12: skani sketch, triangle, and search runtimes on the refseq-rc dataset (4233 complete bacterial genomes) with all-to-all comparisons plotted against number of threads used. The dashed black line indicates perfect $1/t$ scaling where t is the number of threads. Sketching scaling is limited by disk IO. Triangle scaling is limited by disk IO (reading the sketches into memory), and generating the inverted index; note that triangle runs in < 20 seconds with 32 threads. Search only loads the markers into memory and only loads the full indices if comparisons pass the ANI filter, discarding the full index after the comparison. The IO burden of repeatedly loading and discarding the indices causes search to scale relatively poorly with the number of threads compared to the other commands.

References

1. Sokal, R. R. & Rohlf, F. J. The Comparison of Dendrograms by Objective Methods. *Taxon* **11**, 33–40 (1962). 1217208.
2. Robinson, D. F. & Foulds, L. R. Comparison of phylogenetic trees. *Mathematical Biosciences* **53**, 131–147 (1981).
3. Pasolli, E. *et al.* Extensive Unexplored Human Microbiome Diversity Revealed by Over 150,000 Genomes from Metagenomes Spanning Age, Geography, and Lifestyle. *Cell* **176**, 649–662.e20 (2019).
4. Nishimura, Y. & Yoshizawa, S. The OceanDNA MAG catalog contains over 50,000 prokaryotic genomes originated from various marine environments. *Scientific Data* **9**, 305 (2022).
5. Olm, M. R. *et al.* Consistent Metagenome-Derived Metrics Verify and Delineate Bacterial Species Boundaries. *mSystems* **5**, e00731–19 (2020).
6. Alexander, H. *et al.* Eukaryotic genomes from a global metagenomic dataset illuminate trophic modes and biogeography of ocean plankton (2022).
7. Delmont, T. O. *et al.* Functional repertoire convergence of distantly related eukaryotic plankton lineages abundant in the sunlit ocean. *Cell Genomics* **2**, 100123 (2022).
8. Nayfach, S. *et al.* A genomic catalog of Earth's microbiomes. *Nature Biotechnology* **39**, 499–509 (2021).
9. Jain, C., Rodriguez-R, L. M., Phillippy, A. M., Konstantinidis, K. T. & Aluru, S. High throughput ANI analysis of 90K prokaryotic genomes reveals clear species boundaries. *Nature Communications* **9**, 5114 (2018).
10. Parks, D. H. *et al.* A standardized bacterial taxonomy based on genome phylogeny substantially revises the tree of life. *Nature Biotechnology* **36**, 996–1004 (2018).
11. Parks, D. H. *et al.* Recovery of nearly 8,000 metagenome-assembled genomes substantially expands the tree of life. *Nature Microbiology* **2**, 1533–1542 (2017).
12. Pedregosa, F. *et al.* Scikit-learn: Machine learning in Python. *Journal of Machine Learning Research* **12**, 2825–2830 (2011).
