# Supplementary material for: Fast and robust metagenomic sequence comparison through sparse chaining with skani
Source: Nat Methods. 2023 Sep 21;20(11):1661–5. doi: 10.1038/s41592-023-02018-3 (PMC10630134; doi:10.1038/s41592-023-02018-3)
Supplement: Supplementary file 2 — Reporting Summary [file 41592_2023_2018_MOESM2_ESM.pdf]

nature portfolio

Corresponding author(s): Yun William Yu

Last updated by author(s): Jun 23, 2023

Reporting Summary

Nature Portfolio wishes to improve the reproducibility of the work that we publish. This form provides structure for consistency and transparency in reporting. For further information on Nature Portfolio policies, see our Editorial Policies and the Editorial Policy Checklist.

Statistics

For all statistical analyses, confirm that the following items are present in the figure legend, table legend, main text, or Methods section.

n/a	Confirmed	
☐	☒	The exact sample size (*n*) for each experimental group/condition, given as a discrete number and unit of measurement
☐	☒	A statement on whether measurements were taken from distinct samples or whether the same sample was measured repeatedly
☒	☐	The statistical test(s) used AND whether they are one- or two-sided<br>*Only common tests should be described solely by name; describe more complex techniques in the Methods section.*
☒	☐	A description of all covariates tested
☐	☒	A description of any assumptions or corrections, such as tests of normality and adjustment for multiple comparisons
☐	☒	A full description of the statistical parameters including central tendency (e.g. means) or other basic estimates (e.g. regression coefficient) AND variation (e.g. standard deviation) or associated estimates of uncertainty (e.g. confidence intervals)
☒	☐	For null hypothesis testing, the test statistic (e.g. *F*, *t*, *r*) with confidence intervals, effect sizes, degrees of freedom and *P* value noted<br>*Give P values as exact values whenever suitable.*
☒	☐	For Bayesian analysis, information on the choice of priors and Markov chain Monte Carlo settings
☒	☐	For hierarchical and complex designs, identification of the appropriate level for tests and full reporting of outcomes
☐	☒	Estimates of effect sizes (e.g. Cohen's *d*, Pearson's *r*), indicating how they were calculated

Our web collection on statistics for biologists contains articles on many of the points above.

Software and code

Policy information about availability of computer code

Data collection	No software was used.
Data analysis	Our software skani v0.1.0 (described in manuscript and open-sourced at https://github.com/bluenote-1577/skani) was used for data analysis, along with the open source Python scikit-learn 0.22.1 package. Benchmarked against Mash v2.3, FastANI v1.33, sourmash v4.5, OrthoANIu (v1.2), ANIm pyani (v0.2.12).

For manuscripts utilizing custom algorithms or software that are central to the research but not yet described in published literature, software must be made available to editors and reviewers. We strongly encourage code deposition in a community repository (e.g. GitHub). See the Nature Portfolio guidelines for submitting code & software for further information.

Data

Policy information about availability of data

All manuscripts must include a data availability statement. This statement should provide the following information, where applicable:

- Accession codes, unique identifiers, or web links for publicly available datasets
- A description of any restrictions on data availability
- For clinical datasets or third party data, please ensure that the statement adheres to our policy

All data sets are specified in Supplementary Table 2 of the manuscript.

Human research participants

Policy information about studies involving human research participants and Sex and Gender in Research.

Reporting on sex and gender	N/A. (not human research)
Population characteristics	N/A. (not human research)
Recruitment	N/A. (not human research)
Ethics oversight	N/A. (not human research)

Note that full information on the approval of the study protocol must also be provided in the manuscript.

Field-specific reporting

Please select the one below that is the best fit for your research. If you are not sure, read the appropriate sections before making your selection.

☒ Life sciences ☐ Behavioural & social sciences ☐ Ecological, evolutionary & environmental sciences

For a reference copy of the document with all sections, see nature.com/documents/nr-reporting-summary-flat.pdf

Life sciences study design

All studies must disclose on these points even when the disclosure is negative.

Sample size	No sample-size calculation was performed. This is a software/benchmarking paper where we used fixed sample sizes to illustrate the relative performance against baselines of our and prior software. Sample sizes were chosen based on those used for previous studies and what was feasible for our computing platforms.
Data exclusions	No data were excluded.
Replication	As a methods and benchmarking paper, we were primarily interested in measuring the accuracy of out software. Thus, we repeated our analyses on multiple disparate data sets against gold-standard benchmarks. For data set generalizability, we ran on four different benchmark data sets and verified in the manuscript that the results were consistent.<br><br>Additioally, for software replicability, we ran our software on several different compute platforms, including a local machine, and on multiple Google Cloud instances. In every case, our software produced identical results.
Randomization	Not applicable, as we are designing a new ANI computation method, and all software was run on all datasets to compare.
Blinding	Not applicable, as this was a methods and benchmarking software design paper.

Reporting for specific materials, systems and methods

We require information from authors about some types of materials, experimental systems and methods used in many studies. Here, indicate whether each material, system or method listed is relevant to your study. If you are not sure if a list item applies to your research, read the appropriate section before selecting a response.

Materials & experimental systems

n/a	Involved in the study	
☒	☐	Antibodies
☒	☐	Eukaryotic cell lines
☒	☐	Palaeontology and archaeology
☒	☐	Animals and other organisms
☒	☐	Clinical data
☒	☐	Dual use research of concern

Methods

n/a	Involved in the study	
☒	☐	ChIP-seq
☒	☐	Flow cytometry
☒	☐	MRI-based neuroimaging
